# The imprint of microfibres in southern European deep seas

**DOI:** 10.1371/journal.pone.0207033

**Published:** 2018-11-05

**Authors:** Anna Sanchez-Vidal, Richard C. Thompson, Miquel Canals, William P. de Haan

**Affiliations:** 1 GRC Geociències Marines, Departament de Dinàmica de la Terra i de l’Oceà, Universitat de Barcelona, Barcelona, Spain; 2 Marine Biology and Ecology Research Centre, Plymouth University, Plymouth, United Kingdom; University of California, Merced, UNITED STATES

## Abstract

Pollution of the marine environment by large and microscopic plastic fragments and their potential impacts on organisms has stimulated considerable research interest and has received widespread publicity. However, relatively little attention has been paid to the fate and effects of microplastic particles that are fibrous in shape, also referred as microfibres, which are mostly shed from synthetic textiles during production or washing. Here we assess composition and abundance of microfibres in seafloor sediments in southern European seas, filling gaps in the limited understanding of the long-range transport and magnitude of this type of microplastic pollution. We report abundances of 10–70 microfibres in 50 ml of sediment, including both natural and regenerated cellulose, and synthetic plastic (polyester, acrylic, polyamide, polyethylene, and polypropylene) fibres. Following a shelf-slope-deep basin continuum approach, based on the relative abundance of fibres it would appear that coastal seas retain around 33% of the sea floor microfibres, but greater quantities of the fibres are exported to the open sea, where they accumulate in sediments. Submarine canyons act as preferential conduits for downslope transport of microfibres, with 29% of the seafloor microfibres compared to 18% found on the open slope. Around 20% of the microfibres found had accumulated in the deep open sea beyond 2000m of water depth. The remoteness of the deep sea does not prevent the accumulation of microfibres, being available to become integrated into deep sea organisms.

## Introduction

Although already mentioned in the published literature in the early 1990s [1], the term microplastic has been increasingly used since 2004 to describe plastic particles of a few mm in size in the marine environment [2]. Microplastics derive from fragmentation of larger plastic items entering by rivers, sewage, beach littering, runoff, tides and winds [3, 4], and also by direct release of small particles such as plastic pellets [5], cosmetic microbeads [6] and clothing microfibres [7]. Plastic has been released to the marine environment since the 1930s, and is now ubiquitous in the oceans. Plastic debris have been reported in surface and subsurface waters [8, 9, 10], in the seafloor from the shoreline [11, 12] to the deep sea [13, 14] and in polar ice caps [15]. The accumulation of microplastics in the marine environment, and the many questions concerning their distribution and implications for marine wildlife and human health, has recently raised public awareness. Intensive research on this topic has resulted in a rapidly increasing number of publications (see for instance [16]). Consequently, policy recommendations have been provided too to tackle this emerging environmental problem (e.g. [17]).

Microfibres are among the most prevalent type of microplastics observed in the marine environment [11, 18]. However, despite microfibres are highly visible, brightly colored and stand out against fine grained sediments or marine aggregates, only a few studies dealing specifically on plastic microfibre pollution in the marine environment have been published to date. Synthetic (polyester, acrylic, polypropylene, polyamide) microfibres may be entering the ocean via wastewaters [11, 19, 20] and atmospheric fallout [21], and have been found in surface waters [22, 23], sea ice [15] and in coastal [24, 25, 26] and deep water sediments [14, 27]. It has been already shown that microfibres are ingested by pelagic [28, 29, 30] and benthic coastal organisms [25, 31, 32, 33]. The recent discovery of microfibre ingestion and internalization by deep-sea organisms in a natural setting by [34] has underlined the need to quantify of this human waste in the deep marine environment.

**Fig 1:**
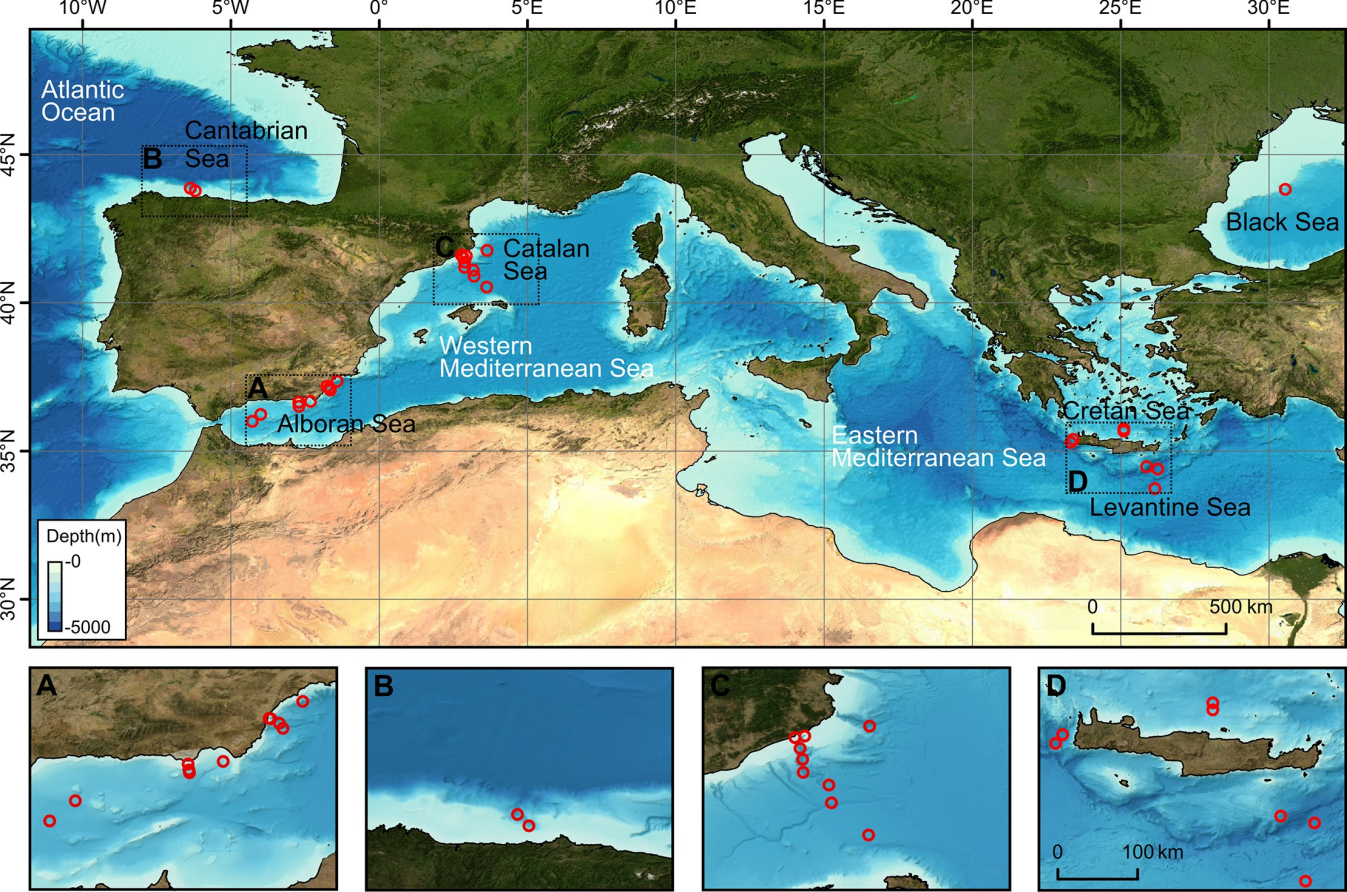
General map of the southern European seas. The location of surface sediment sampling stations for the analysis of plastic fibres is shown as red open circles. Black boxes mark areas that are shown as zoom-ins below. The map was generated using the GEBCO_2014 grid (http://www.gebco.net/data_and_products/gridded_bathymetry_data/) and ArcGIS version 10.3 (http://desktop.arcgis.com/en/arcmap/).

Given the particularly high concentration of microplastics found in surface waters of the Mediterranean Sea [35, 36], microfibre quantification in sediments is required to confirm or dismiss the relative importance of the deep sea as a microplastics sink. Indeed the landlocked character and limited outflow of surface waters of the Mediterranean Sea, its densely populated coastline including seasonal tourist peaks and intensive fishing, shipping and other industrial activities, made it candidate to be the sixth great floating microplastic accumulation zone after the five subtropical gyres [35, 37]. Here we present new data on the distribution of plastic microfibres after a widespread survey of seabed sediments in southern European seas including the northeast Atlantic Ocean (Cantabrian Sea), the Mediterranean Sea (Alboran Sea, Catalan Sea, Cretan Sea and Levantine Sea) and the Black Sea at depths from 42 m at the continental shelf to 3,500 m in the abyssal plain (Fig 1). Such a wide depth range allowed investigating patterns of microfibre distribution along the coastal-deep sea continuum.

## Materials and methods

Seabed sediment samples were obtained using either a multicorer or a Van Veen grab at 29 stations in the southern European seas (Fig 1) during 10 oceanographic cruises, from 2009 to 2015. Once on deck, the first cm of each core/grab sample was immediately subsampled to minimize exposure to the air and stored in a clean aluminium container or a polyethylene zip-lock plastic bag and kept in a cold dry place. No specific permission was required for collecting the sediment samples for this study, as most of the activities were carried out in waters beyond national jurisdiction, except for some shallower continental shelf stations, where appropriate permissions were inherent to research projects funded by the Greek and Spanish governments. None of the field studies involved endangered or protected species.

Microfibre extraction was performed in the designated clean Microplastic Laboratory at the University of Plymouth. The laboratory was thoroughly cleaned daily, access restricted and 100% cotton muslin placed as a flap over the door opening. All precautions were taken to avoid contamination of samples, including repeatedly rinsing the used equipment with ultrapure water, using only glass material covered with aluminium foil, wearing 100% natural fibre laboratory coat and clothing, and scrubbing hands and nails regularly. Daily records of air pollution were made and no synthetic microfibres were seen.

Plastic microfibres were extracted from 10 ml of sediment using 200 ml of saturated sodium chloride (NaCl) solution (1.2 g cm^-3^) [2]. Three sequential extractions were performed using a glass fibre filter to separate suspended debris from settled sediment components after shaking for 30 seconds and settling for 5 minutes. This process was repeated 3 times for every sample. Blanks were run every 2 samples and did not indicate any source of potential contamination. As expected due to the low volume of sample used [38], all microplastics found were microfibers.

We report on plastic microfibres in 50 ml of sediment (MF50 from here onwards) for ease of comparison with other environmental matrices (e.g. ice and water) and other studies [14]. MF50 converts to microfibres per 50 grams of dry weight assuming an average sediment dry density of 1 g cm^-3^ (based on own data) and to microfibres per unit of area assuming a 1cm-thick sediment layer.

Since the density of the saturated NaCl solution is only 1.2 g ml^-1^, high-density fibres such as polyester or cellulose, and regenerated cellulose, may not float. However, we found that those fibres were efficiently extracted from the sediments. Fibre adherence to air bubbles formed when vigorously shaking the saturated NaCl solution with the sediment sample may have subsequently conveyed the fibres to the surface of the supernatant, according to the Stoke’s Law, from where they were then filtrated. Indeed dense fibre separation by flotation due to the action of air microbubbles have been widely used in mining and paper and pulp industries. Air flotation as a tertiary treatment in wastewater effluents has also been proven to efficiently remove 95% of the microfibers [39].

All filters were examined under a binocular microscope, and any debris that was of unnatural appearance was transferred to sealed containers and subsequently identified by spectrometry. A Vertex 70 Fourier transform infrared (FT-IR) spectrometer at the University of Plymouth was used for polymer identification of each microfibre found. The spectra obtained were compared to a spectral database of synthetic polymer (Bruker I26933 Synthetic fibres ATR library). Bruker’s Opus spectroscopy software was used for measurement, processing and evaluation of the IR spectra. All data generated during this study are included in S1 Table.

The normality of the data was tested using the Kolmogorov-Smirnov test before performing a one-way ANOVA to explain the differences between number of microfibres in the investigated seas (i.e. Cantabrian Sea, Alboran Sea, Catalan Sea, Cretan Sea, Levantine Sea and Black Sea) and geomorphic environments (i.e. continental shelf, open continental slope, submarine canyons and deep basin). Statistical significance of the data was assumed when p < 0.05.

The grain size distribution of surface sediments was determined using a Coulter LS230 Laser Diffraction Particle Size Analyzer. Organic matter in the samples was first oxidized with 10% H_2_O_2_.

## Results and discussion

A total of 202 microfibres were found in the 29 surface sediment samples analysed. Fibres length varied between 3 and 8 mm, and the most abundant colours were red (27%), white (23%), blue (21%) and black (19%). Fibre abundance ranged between 10 and 70 MF50 and averaged 34.8 ± 18.3 MF50, which is equivalent to 6,965 ± 3,669 microfibres m^-2^. Microfibres abundances are of the same order of magnitude as those reported in deep-sea sediments in the subpolar North Atlantic, the NE Atlantic and the SW Indian oceans [14], and significantly more abundant (2 to 8 orders of magnitude larger) than floating fibres in ocean surface and subsurface waters [22, 23, 40]. Even though low floating microfibre abundances may be directly related to the relatively large (usually 330 μm) mesh size of the net [41], this is new evidence, after [14], confirming deep-sea sediments as a major sink for microfibres. The fibres identified by spectrometry included cellulose (79.7%), polyester (polyethylene terephthalate) (12.9%), acrylic (polymethyl methacrylate) (4.5%), polyamide (1.0%), polyethylene (1.0%), and polypropylene (1.0%) (S1 Table).

The main type of microfibre found in seafloor sediments was thus essentially not plastic but cellulose fibres, that consisted of both dyed natural cellulose (cotton, linen) and manufactured fibres composed of regenerated cellulose, e.g. rayon. Rayon is a man-made fibre produced from dissolving cellulose-based raw material, an industrial process that requires an intensive use of water and energy, and extensive insidious toxic chemical treatment [42]. Because the chemical composition and properties of the natural polymer is significantly modified during the manufacturing process, rayon has been generally considered when reporting man-made microfibres [14, 22, 43]. The main uses of both natural and regenerated cellulose fibre are clothing and apparel, industrial textiles such as mechanical rubber goods, and feminine hygiene products. Natural and regenerated cellulose fibres have been recently found in atmospheric fallout [21], rivers [44], macrofauna [45] and fish [46, 47, 48].

The second most abundant fibre was polyester, which is the most used synthetic fibre worldwide [49]. Because of its high resistance, polyester is utilized in all types of clothing, especially high-performance outdoor wear and home furnishings. The third most abundant fibre was acrylic, which is usually blended with wool and mostly used in clothing and home furnishings too. Then we found polyamide (i.e. nylon), which is used in clothing, home furnishing and industrial products such as fishing gear because of its lightness and resilience, and polyethylene and polypropylene, which are the lightest fibres, often used in sportswear due to their high resistance and moisture repellence.

These extremely thin (less than 0.1 mm in diameter) but resilient fibres are mostly discharged into wastewater from domestic washing machines [11, 18], each garment producing between 1,900 and 700,000 fibres [19, 18]. In the last decades, the use of synthetic polymer fibres by the clothing industry has overtaken that of natural cotton. Declining cotton production year-on-year, the price and properties of synthetic fibres (i.e. resistance, moisture-wicking), and the growing demand for clothing, have made plastic fibres more desirable to manufacturers. Accordingly, a direct link between washing clothes and marine pollution can be established based on the similar proportions of the plastic polymer found in textiles (polyester > polyamide > polypropylene > acrylic) [49], sewage (polyester > polyamide > acrylic) [18, 50], coastal habitats (polyester > acrylic > polyamide > polypropylene) [51], and the deep sea floor (polyester > acrylic > polyamide) (this study). Furthermore, the atmospheric compartment should not be neglected as an additional mode of microfibre spreading, as a similar proportion of cellulose and polyester fibres have been recently observed in the atmospheric fallout in an urban environment [21].

Our results show the dominance of cellulosic fibres over synthetic polymers. In contrast, synthetic fibres dominate the global fibre market, with 65% of the share, while natural and man-made cellulosic fibres altogether comprise only a 35% [52]. Shedding of fibres is a relatively new concept in textile development [53], and, to our knowledge, no studies have yet investigated microfibre shedding from cellulose vs. plastic textiles. Assuming a roughly equivalent release of fibres of each polymer to the aquatic environment, data suggest that polymer density is the key component controlling the spreading of microfibres to the deep.

Despite sedimentation of nonspherical particles such as fibres is still poorly understood, and may depend on drag forces on the different shapes and curvature of the fibres [54], cellulosic fibres are significantly denser than seawater and are thus more likely to sink. Accordingly, cellulose is found in large quantities in deep-sea sediments, reaching up to 27.9 MF50 (Table 1). Polyester is also denser than seawater and, consequently, is also found in high quantities in the deep sea, with up to 4.5 MF50 ml. After being injected into the marine environment from sewage disposal sites and wastewater treatment plants [55], suspended high-density microfibres may be able to settle out from the freshwater plume when the flow rate decreases and microfibre buoyancy becomes negative. Then, fibres may behave as very fine grained sediments, being repeatedly resuspended and transported towards the shelf edge and downslope by storm-induced turbidity currents, dense shelf water cascading, hyperpycnal flows or internal waves [56, 57]. In contrast, low-density microfibres such as polyethylene and polypropylene may float and sink only when negative buoyancy is reached due to ballast effect by e.g. colonization by organisms, adherence to phytoplankton and/or aggregation with organic debris [14, 58]. Accordingly, polyethylene and polypropylene are the most abundant compounds found afloat in Mediterranean Sea waters (68% of all particles [36]). Very low abundances of these two low-density polymers (<2%) in the deep sea support our view (Table 1), which, by the way, would have settled due to biofouling [58].

**Table 1:** Densities and abundances of microfibres.

Microfibre polymer, and natural and laboratory solutions	Density (g cm^-3^)	Abundance (MF50)
Polypropylene	0.90	0.3
Polyethylene	0.95	0.3
**Seawater**	**1.02**	**—**
Polyamide	1.16	0.3
Acrylic	1.20	1.6
**Saturated NaCl solution**	**1.20**	**—**
Polyester	1.37	4.5
Regenerated cellulose	1.44	27.9
Natural cellulose	1.50	

Densities of the different types of polymers extracted from surface sediment layers of the southern European seas, along with those of seawater and the hypersaline (saturated) solution used for microfibre extraction, and abundance of each polymer in the analysed sediments. Fibres denser than the saturated NaCl solution were recovered because they attach to raising air bubbles formed when shaking the solution in the laboratory (see Methods section). MF50: microfibres in 50 ml of sediment.

Very little is known about the fate of plastic debris in the marine environment. The degradation of polyester, polyamide, polyethylene and polypropylene occurs primarily through thermo- and UV-induced reactions [59, 60, 61]. Therefore, once microfibres sink in the deep sea the rate of degradation may decrease dramatically [60]. However, there are some evidences that microbes may also play a role via physical or metabolic means [62, 63]. The time required to completely degrade to CO_2_ plastics is estimated to be on the order of hundreds to thousands of years [64]. Even less is known about the degradation of natural and regenerated cellulose in the marine environment. It has been shown that biodegradability of regenerated cellulose is higher than that of natural cellulose [65], or that dyed fibres are somehow protected from to microbial degradation [66]. However it is currently unknown the durability of cellulosic material in the deep sea.

**Fig 2:**
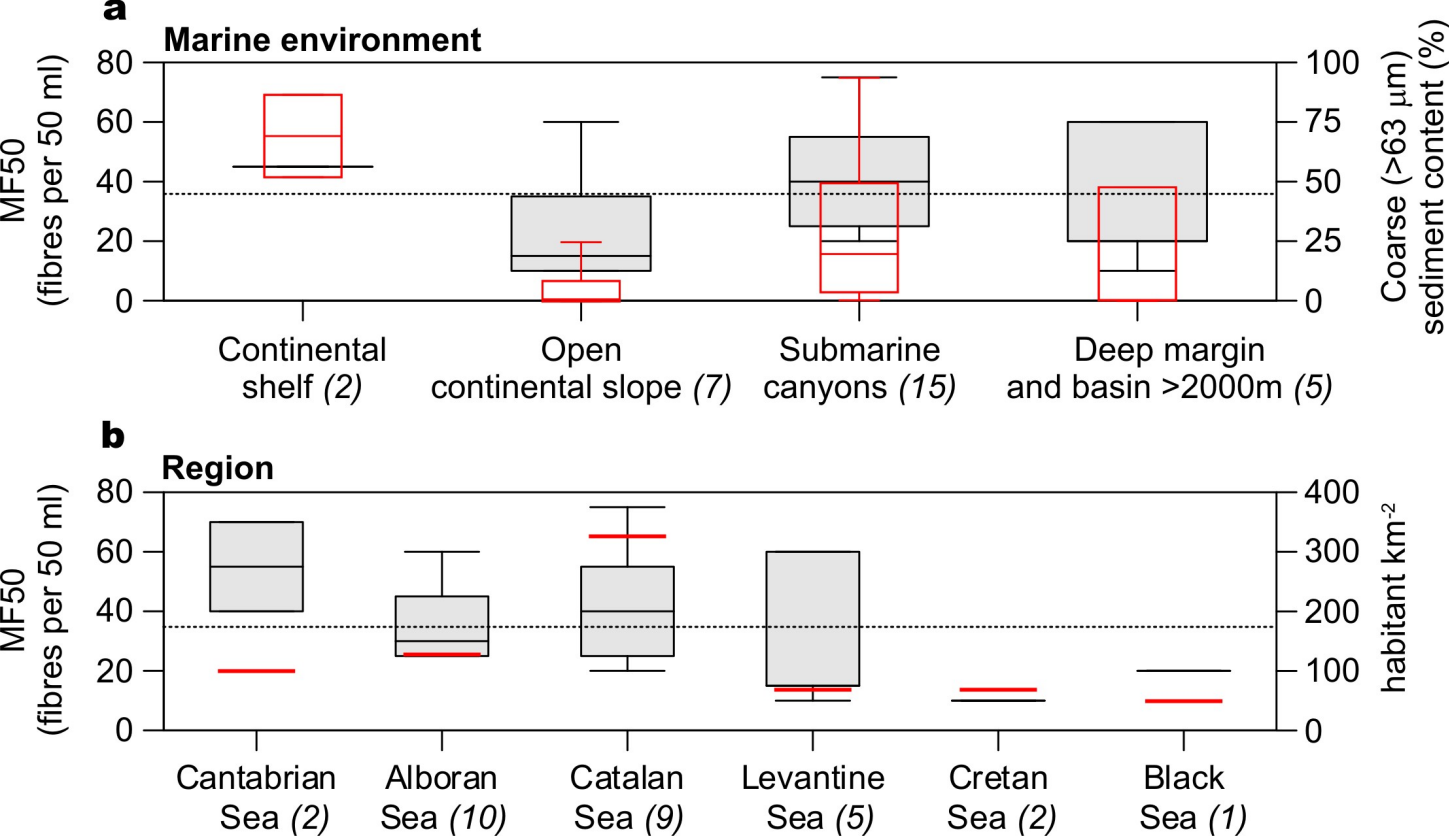
**Boxplot showing microfibres found per 50 ml of sediment (MF50) per marine environment (a) and region (b).** Volume percentage of sediments > 63 μm and human population per square kilometer in the adjacent continental landmass [67] are also shown. The caps at the end of each box indicate the extreme values. The box is defined by the lower and upper quartiles, and the line in the center of the box is the median. The dotted line shows mean microfibre abundance. Number in brackets show the number of samples.

Relative abundance of fibres in different marine environments (i.e. continental shelf, open continental slope, submarine canyons and deep basin) and region (i.e. Cantabrian Sea, Alboran Sea, Catalan Sea, Cretan Sea, Levantine Sea and Black Sea) have been quantified (Fig 2, S1 Table).

There were no statistically significant differences among geomorphic environments (F(3, 25) = 1.87, p = 0.16) or seas (F(5, 23) = 2.17, p = 0.09) as determined by one-way ANOVA, which illustrate the ubiquitous distribution of fibres in the marine environment. However, not achieving a statistically significant result does not mean that there is no spatial variation of microfibre abundance at all. Abundance of microfibres in each marine environment relative to the total abundance in the sea floor show that continental shelf sediments retain 33% of microfibres found in the sea floor. Beyond the shelf edge fibres are found in significantly different proportions in submarine canyons (29%) and open slopes (18%). Predominance of coarse surface sediments in shelf and, to a lesser extent, submarine canyon environments, show the preferential dispersal pathway for the sand-sized material that moves from the shelf and that ultimately may end up in the deep sea (Fig 2). Supported by many references on the important role of submarine canyons as main conduits for sediment transport to the deep sea [56, 68, 69], this would suggest that are also preferential conduits for microfibres transport. Furthermore, the role of canyons in carrying microfibres and other pollutants to the deep is tremendously reinforced when they are the loci of highly dynamic shelf to basin export processes as those occurring in the NW Mediterranean Sea, which result from intense coastal storms and dense shelf water cascading [70, 71]. These oceanographic processes cause concentrations of organochlorine compounds, polybrominated diphenyl ethers, perfluoroalkyl substances and anthropogenic metals in deep-water sediments of the NW Mediterranean Sea that are amongst the highest recorded in the marine environment [72, 73, 74, 75]. Finally, 20% of the microfibres accumulate in the deep sea beyond 2,000 m of water depth.

The highest fibre densities are found in the Cantabrian Sea, followed by the Catalan Sea and the Alboran Sea in the NW and SW Mediterranean Sea, respectively, while the lowest densities are found in the Eastern Mediterranean Sea and the Black Sea. Despite reasonable fit to human population density in the adjacent continental landmass (Fig 2), accumulation of microfibers in the deep sea may be mainly related to the prevailing oceanographic conditions and the presence of active sediment transport processes.

A recent study has provided for the first time evidence of microfibres being ingested by deep-sea organisms in a natural setting [34]. Microfibres of acrylic, polypropylene, rayon and polyester were found inside benthic organisms of a wide range of taxa from phyla Cnidaria, Echinodermata and Arthropoda at 334 to 1795 m of water depth in the Equatorial mid-Atlantic and the SW Indian oceans. However, the long-term impact of microfibre ingestion on deep-sea organisms is yet to be determined and probably depends on many factors including type of polymer and abundance of microfibres in the deep-sea floor [14], capacity to adsorb harmful chemical substances [76, 77], as well as organism physiology and ecology [34]. This applies not only to plastic microfibres but also to cellulose fibres, which associated dyes or additives could also be potentially harmful for the biota [45]. In any case, the persistent nature of microfibres [60] makes evident the need to design effective management strategies for reducing emissions to the environment, such as changes in textile composition, washing conditions or filtration of effluents [19].

## Supporting information

S1 Table Details of sampling location and quantity (microfibres per 50 ml of sediment, MF50) and type (polymer) of fibres found. (DOCX)
