# Supplementary material for: The imprint of microfibres in southern European deep seas
Source: PLoS One. 2018 Nov 5;13(11):e0207033. doi: 10.1371/journal.pone.0207033 (PMC6218086; doi:10.1371/journal.pone.0207033)
Supplement: S1 Table — (DOCX) [file pone.0207033.s001.docx]

**S1 Table**. Details of sampling location and quantity (microfibres per 50 ml of sediment, MF50) and type (polymer) of fibres found.

| **Station** | **Environment** | **Depth** | **Latitude** | **Longitude** | **Year** | **Cellulose** | **Polyester** | **Acrylic** | **Polyethylene** | **Polypropylene** | **Polyamide** | **Total** |
| --- | --- | --- | --- | --- | --- | --- | --- | --- | --- | --- | --- | --- |
|  |  | **(m)** |  |  |  |  |  |  |  |  |  | **(MF50)** |
| **Cantabrian Sea** |  |  |  |  |  |  |  |  |  |  |  |  |
| DMAC1200 | Submarine canyon | 1,207 | 43º 46.52'N | 6º 12.78'W | 2012 | 65 | 0 | 0 | 0 | 5 | 0 | **70** |
| DMAC2000 | Submarine canyon | 2,000 | 43º 54.24'N | 6º 20.53'W | 2012 | 30 | 10 | 0 | 0 | 0 | 0 | **40** |
| **Alboran Sea** |  |  |  |  |  |  |  |  |  |  |  |  |
| NUR2VV1 | Continental shelf | 42 | 36º 39.27'N | 2º 42.70'W | 2015 | 45 | 0 | 0 | 0 | 0 | 0 | **45** |
| NUR2VV2 | Open continental slope | 248 | 36º 35.24'N | 2º 42.19'W | 2015 | 25 | 10 | 0 | 0 | 0 | 0 | **35** |
| NUR2VV4 | Submarine canyon | 389 | 37º 10.29'N | 1º 47.65'W | 2015 | 25 | 0 | 0 | 0 | 0 | 0 | **25** |
| NUR2MC09 | Submarine canyon | 407 | 36º 41.06'N | 2º 18.88'W | 2015 | 30 | 10 | 0 | 5 | 5 | 0 | **50** |
| NUR2VV3 | Open continental slope | 498 | 36º 33.38'N | 2º 41.78'W | 2015 | 60 | 0 | 0 | 0 | 0 | 0 | **60** |
| NUR2VV5 | Submarine canyon | 609 | 37º 10.10'N | 1º 46.53'W | 2015 | 25 | 0 | 0 | 0 | 0 | 0 | **25** |
| HERMC6 | Open continental slope | 770 | 38º 48.67'N | 0º 39.59'E | 2009 | 15 | 10 | 0 | 0 | 0 |  | **25** |
| NUR2VV10 | Submarine canyon | 932 | 37º 21.81'N | 1º 24.82'W | 2015 | 20 | 5 | 0 | 0 | 0 | 0 | **25** |
| NUR2MC12 | Submarine canyon | 1,475 | 37º 07.03'N | 1º 40.76'W | 2015 | 45 | 0 | 0 | 0 | 0 | 0 | **45** |
| NUR2MC13 | Submarine canyon | 1,857 | 37º 03.54'N | 1º 38.27'W | 2015 | 25 | 0 | 0 | 0 | 0 | 0 | **25** |
| **Catalan Sea** |  |  |  |  |  |  |  |  |  |  |  |  |
| P3III | Continental shelf | 67 | 41º 38.53'N | 2º 47.35'E | 2009 | 10 | 20 | 15 | 0 | 0 | 0 | **45** |
| BC300III | Submarine canyon | 281 | 41º 39.57’N | 2º 54.05’E | 2009 | 30 | 20 | 0 | 0 | 0 | 5 | **55** |
| BC1200III | Submarine canyon | 1,190 | 41º 31.17'N | 2º 50.84'E | 2012 | 15 | 10 | 0 | 0 | 0 | 0 | **25** |
| PROM MC05 | Submarine canyon | 1,667 | 41º 23.72'N | 2º 52.60'E | 2011 | 30 | 0 | 5 | 0 | 0 | 0 | **35** |
| BC2000 | Submarine canyon | 1,980 | 41º 14.90'N | 2º 52.97'E | 2012 | 45 | 5 | 0 | 0 | 0 | 5 | **55** |
| PROM MC18 | Submarine canyon | 2,064 | 41º 46.30'N | 3º 38.14'E | 2011 | 70 | 5 | 0 | 0 | 0 | 0 | **75** |
| HERMCMR1 | Deep margin and basin | 2,117 | 40º 32.31'N | 3º 37.40'E | 2009 | 15 | 0 | 5 | 0 | 0 | 0 | **20** |
| BC2200 | Submarine canyon | 2,196 | 41º 06.30'N | 3º 10.44'E | 2012 | 40 | 0 | 0 | 0 | 0 | 0 | **40** |
| OS2200 | Deep margin and basin | 2,222 | 40º 54.20'N | 3º 12.19'E | 2012 | 20 | 0 | 0 | 0 | 0 | 0 | **20** |
| **Cretan Sea** |  |  |  |  |  |  |  |  |  |  |  |  |
| RED5 | Open continental slope | 991 | 34º 36.00'N | 24º 8.40'E | 2010 | 10 | 0 | 0 | 0 | 0 | 0 | **10** |
| RED4A | Open continental slope | 1,018 | 35º 40.80'N | 25º 6.00'E | 2010 | 10 | 0 | 0 | 0 | 0 | 0 | **10** |
| **Levantine Sea** |  |  |  |  |  |  |  |  |  |  |  |  |
| RED3.1 | Open continental slope | 1,615 | 35º 45.60'N | 25º 6.00'E | 2010 | 5 | 5 | 5 | 0 | 0 | 0 | **15** |
| BF22 | Open continental slope | 2,015 | 34º 28.67'N | 25º 52.14'E | 2009 | 10 | 0 | 0 | 5 | 0 | 0 | **15** |
| RED2A | Deep margin and basin | 2,720 | 33º 42.90'N | 26º 20.40'E | 2010 | 5 | 5 | 0 | 0 | 0 | 0 | **10** |
| RED3 | Deep margin and basin | 3,314 | 35º 18.00'N | 23º 19.20'E | 2010 | 45 | 5 | 10 | 0 | 0 | 0 | **60** |
| REDECO11 | Deep margin and basin | 3,500 | 34º 23.98'N | 26º 15.05'E | 2011 | 15 | 10 | 5 | 0 | 0 | 0 | **30** |
| **Black Sea** |  |  |  |  |  |  |  |  |  |  |  |  |
| GHASSMTB01 | Submarine canyon | 840 | 43º 50.16'N | 30º 34.27'E | 2015 | 20 | 0 | 0 | 0 | 0 | 0 | **20** |
